# Incidental thyroid carcinoma in surgically treated multinodular goiter: a retrospective study

**DOI:** 10.3389/fsurg.2026.1825352

**Published:** 2026-07-02

**Authors:** G. Pavone, E. Lamanna, M. Pacilli, E. Khoury, A. Ambrosi, N. Tartaglia

**Affiliations:** Department of Medical and Surgical Sciences, University of Foggia, Foggia, Italy

**Keywords:** endocrine surgery, incidental thyroid cancer, multinodular goiter, thyroid hormone, total thyroidectomy

## Abstract

**Background:**

Multinodular goiter (MNG) is a common thyroid disorder frequently encountered in clinical practice. Although it is generally considered a benign condition, thyroid carcinoma may occasionally be detected in patients undergoing surgery for MNG. The reported prevalence of malignancy in this setting varies widely across different populations and clinical series. The present study aimed to evaluate the incidence of incidental thyroid carcinoma in patients undergoing surgical treatment for multinodular goiter and to analyze the associated demographic and clinicopathological characteristics.

**Materials and methods:**

This retrospective study included patients with a preoperative diagnosis of multinodular goiter who underwent total or partial thyroidectomy between January 2018 and May 2024 at the Department of Medical and Surgical Sciences, University Hospital “Ospedali Riuniti” of Foggia. Clinical, demographic, and pathological data were collected from medical records. Statistical analyses were performed to explore potential associations between patient characteristics and the occurrence of incidental thyroid carcinoma.

**Results:**

A total of 223 patients were included in the study. Incidental thyroid carcinoma was identified in 32 cases, corresponding to an overall incidence of 14.4%. Among these patients, 24 were female (75%) and 8 were male (25%), with a female-to-male ratio of 3:1. The incidence of carcinoma was 13.3% in female patients and 19% in male patients, although this difference did not reach statistical significance (*χ*^2^ = 0.929; *p* = 0.335). Papillary thyroid carcinoma represented the most common histological subtype, accounting for 78.1% of cases. Patients diagnosed with carcinoma were significantly younger than those without malignancy (mean age 49.4 ± 16.7 vs. 56.2 ± 13.1 years; *p* = 0.008). Logistic regression analysis showed that increasing age was associated with a progressive reduction in the probability of malignancy (OR=0.963; *p* = 0.009).

**Conclusion:**

Incidental thyroid carcinoma represents a relatively frequent finding in patients undergoing surgery for multinodular goiter. Papillary carcinoma is the predominant histological subtype, and no significant sex-related differences were observed. Although variables such as age and thyroid weight may contribute to risk stratification, the current evidence remains insufficient to support modifications to existing screening strategies for thyroid nodules.

**Trial registration:**

This article was retrospectively registered on the 10th of April 2025. The UIN for ClinicalTrial.gov Protocol Registration and Results System is: NCT06924242 for the Organization UFoggia (https://clinicaltrials.gov/ct2/show/NCT06924242).

## Background

Thyroid cancer is the most frequent malignancy of the endocrine system and has been widely investigated because of its steadily increasing incidence worldwide and its heterogeneous clinical presentation.

Among thyroid disorders, multinodular goiter (MNG) is commonly encountered in clinical practice. Its prevalence varies considerably depending on iodine availability, ranging from approximately 0.4% to 7.2% in iodine-sufficient areas and reaching 25%–33% of the population in regions with iodine deficiency (1). MNG is characterized by an enlarged thyroid gland containing multiple nodules and is believed to arise from the interaction of genetic susceptibility, environmental factors, and dietary influences.

Although multinodular goiter has traditionally been considered a benign condition, several studies have demonstrated that thyroid carcinoma may occur within multinodular glands. Reported incidence rates vary widely across the literature, ranging from approximately 5% to 35% depending on the population studied and the diagnostic approach adopted (2). Bove et al. reported an overall incidence of 8.9% in 2023, including 6.1% microcarcinomas and 2.8% clinically relevant thyroid carcinomas (3). In contrast, a study published in 2021 including 3,233 patients identified thyroid carcinoma in 1,026 cases, corresponding to a prevalence of 31.7% (4, 5). In Southern Italy, a study conducted in Calabria—an area characterized by mild to moderate iodine deficiency—reported an overall prevalence of incidental thyroid carcinoma of 12.5% (6).

Such variability may partly reflect genetic differences among populations as well as environmental and lifestyle factors, particularly dietary iodine intake. However, the relationship between iodine consumption and thyroid cancer remains controversial. Following the introduction of salt iodization programs and iodine supplementation, several studies observed an increase in papillary thyroid carcinoma (PTC) incidence accompanied by a reduction in follicular thyroid carcinoma (FTC). Conversely, some meta-analyses have suggested that higher iodine intake may actually be associated with a reduced risk of thyroid cancer (7). Other investigations have reported an association between metabolic factors—such as obesity and excess body weight—and the development of thyroid neoplasms (8, 9). These findings underline the complex interaction between dietary, environmental, and metabolic influences in thyroid tumorigenesis.

The diagnostic assessment of multinodular goiter presents particular challenges because the presence of multiple nodules can complicate the identification of potentially malignant lesions. Historically, the risk of malignancy in MNG was considered lower than in solitary nodules. However, more recent evidence indicates that nodules within MNG that demonstrate progressive growth, become dominant, or undergo structural changes may have a risk of malignancy comparable to that observed in solitary nodules.

Fine-needle aspiration (FNA) cytology remains the principal diagnostic tool used to distinguish nodules with a high probability of malignancy from benign lesions that can be managed conservatively. In solitary nodules, the false-negative rate of FNA is estimated to be approximately 3% (9). In the context of multinodular goiter, however, the diagnostic process may be more complex due to the large number of nodules present and the need to select those most appropriate for biopsy, which may reduce the overall sensitivity of the procedure (10, 11). Ultrasound risk stratification systems such as TIRADS may support clinicians in identifying suspicious nodules and guiding the selection of lesions requiring cytological evaluation.

Recent advances in molecular diagnostics have further contributed to the evaluation of thyroid nodules. Nikiforov et al. (13) described the use of molecular markers—including BRAF and RAS mutations and RET/PTC rearrangements—to improve the prediction of thyroid malignancy. Their study demonstrated that combining molecular testing with conventional diagnostic methods may increase the accuracy of cancer detection in multinodular goiter (12). Similarly, Agrawal et al. (30) highlighted the role of molecular diagnostics in refining risk stratification and clinical management of thyroid nodules (13).

The relationship between gender and thyroid cancer has produced inconsistent findings in the literature. Some studies have reported a higher risk of incidental papillary carcinoma in male patients (4), whereas others have observed a greater prevalence among females (3, 6).

Overall, the available evidence highlights the complex and not yet fully clarified relationship between multinodular goiter and thyroid carcinoma. Although the absolute risk of malignancy within MNG remains relatively limited, accurate diagnostic evaluation is essential for early identification of malignant lesions. The integration of ultrasound imaging, cytological assessment, and molecular testing has improved diagnostic accuracy and may facilitate more appropriate clinical decision-making. Furthermore, the identification of demographic and clinical variables associated with malignancy could help refine risk stratification strategies in patients with multinodular goiter.

The aim of the present study was therefore to evaluate the incidence of incidental thyroid carcinoma in a cohort of patients undergoing surgery for multinodular goiter and to analyze the clinicopathological characteristics associated with malignancy.

## Materials and methods

### Patients

This retrospective study included patients with a preoperative diagnosis of multinodular goiter who underwent total or partial thyroidectomy from January 2018 to May 2024 at the Department of Medical and Surgical Sciences at the University Hospital “Ospedali Riuniti” in Foggia. The study's objective was to investigate the incidence of thyroid carcinoma in patients undergoing thyroidectomy for MNG, also analyzing associated demographic and clinical variables.

The diagnosis of multinodular goiter was established through clinical evaluation and thyroid ultrasound examination.

Inclusion criteria were: (1) patients diagnosed with multinodular goiter via thyroid ultrasound, with or without FNA cytology, (2) patients who underwent surgery, and (3) availability of complete clinical data. Exclusion criteria were: patients with a history of thyroid carcinoma or other thyroid neoplasms, those who did not complete postoperative follow up, and patients with preoperative FNA results indicating malignancy (TIR 4–5), suspicion of malignancy (TIR 3), or indeterminate significance (TIR 1) per the 2014 criteria of the Italian Society of Pathological Anatomy and Cytology (SIAPEC).

Demographic, clinical, and pathological data were collected retrospectively from paper and electronic medical records.

These included age, sex, results of any FNA cytology, type of surgery, weight of the resected thyroid, and definitive histopathological findings.

Fine-needle aspiration biopsy (FNAB) was performed selectively according to ultrasound risk stratification and current clinical guidelines. In patients with multinodular goiter, FNAB was preferentially performed on nodules with suspicious ultrasound features or dominant nodules exceeding 1 cm.

For patients diagnosed with incidental thyroid carcinoma who had undergone preoperative FNAB, a retrospective correlation between cytological sampling and final histopathology was performed. In most cases, malignancy was identified in nodules different from those biopsied, highlighting the limitations of nodule selection in multinodular goiter.

Histopathological diagnoses were classified according to the 5th edition of the World Health Organization (WHO) Classification of Thyroid Tumours (2022).

Therefore, carcinomas detected in these patients were classified as incidental thyroid carcinomas (ITC), defined as malignancies identified only after histopathological examination of thyroidectomy specimens performed for presumed benign disease.

Preoperative ultrasound parameters, such as dominant nodule diameter and total thyroid volume, were not consistently available in the retrospective dataset and were therefore not included in the statistical analysis. For this reason, thyroid weight measured after surgical resection was used as an indirect indicator of gland size

### Statistical analysis

Continuous variables were represented as mean values along with standard deviation (SD), while categorical variables were presented as counts and percentages. Percentages were compared using Pearson's chi-square test, a statistical method employed to assess whether the observed frequency distribution differs significantly from the expected distribution in a given sample. In essence, it evaluates whether two categorical variables are independent.

To investigate potential correlations between continuous variables and the presence of malignancy, an independent samples Student's t-test was conducted. This test determines whether there are statistically significant differences between the means of two groups.

Logistic regression analysis was performed to examine the potential relationship between age and incidental thyroid carcinoma (ITC). Logistic regression is a statistical test used to analyze the relationship between a binary dependent variable (e.g., yes/no, 0/1) and one or more independent variables. This method estimates the impact of independent variables on the dependent variable. The resulting odds ratios (OR) were reported with 95% confidence intervals (CI). A significance level of 0.05 was selected to determine statistical significance. Statistical analysis was conducted using SPSS software.

## Results

The study included a cohort of 223 patients diagnosed with multinodular goiter (MNG) at our center between January 2018 and May 2024. The cohort included 181 females (81.2%) and 42 males (18.8%), with a mean age of 55.3 years (SD 15.5). All cases were benign multinodular goiter, with 151 patients (67.7%) undergoing fine-needle aspiration (FNA) yielding benign results, and 72 patients (32.3%) not undergoing FNA. All patients underwent surgical treatment for symptomatic goiter (compressive symptoms, hypo-/hyperthyroidism), with 202 (90.6%) undergoing total thyroidectomy and 21 (9.4%) undergoing lobectomy (Table 1). Patients diagnosed with incidental carcinoma post-total thyroidectomy were referred directly for endocrinological and oncological follow-up, while those who underwent lobectomy received the same guidance following completion of thyroidectomy.

**Table 1:** Characteristics of the study participants.

Characteristics	Mean (SD) or number (%)
Male, n^o^	42 (18,8%)
Female, n^o^	181 (81,2%)
Age, years	55,3 (±13,5)
Benign FNA (TIR2), n^o^	151 (67,7%)
FNA not done, n^o^	72 (32,3%)
Total thyroidectomy, n^o^	202 (90,6%)
Lobectomy, n^o^	21 (9,4%)
Thyroid weight, grams	68,4 (±57,9)

The choice between lobectomy and total thyroidectomy was based on clinical and imaging criteria. Lobectomy was generally performed in patients with unilateral dominant nodules, absence of suspicious features in the contralateral lobe, and lower thyroid volume. Total thyroidectomy was preferred in cases of bilateral disease, compressive symptoms, hyperthyroidism, or large goiters. In selected cases, the decision was influenced by surgeon preference and intraoperative findings.

Out of the 223 patients, 32 cases of thyroid carcinoma were diagnosed, corresponding to an incidence rate of 14.4% (Table 2). Among these, 24 were female (75%) and 8 were male (25%), with a female-to-male ratio of 3:1. Thyroid carcinoma was identified in 13.3% of all females and 19% of all males. Although a difference in incidence between the sexes was observed, it was not statistically significant (chi=0.929; *p* = 0.335).

**Table 2:** Characteristics of patient with carcinoma.

Characteristics	Mean (SD) or number (%)
Carcinoma, n^o^	32 (14,4%)
M	8 (25%)
F	24 (75%)
Benign neoplasms, n^o^	22 (10,9%)
Age, years	49,4 (±16,7)
Benign FNA (TIR2), n^o^	18 (56,3%)
FNA not done, n^o^	18 (56,3%)
Total thyroidectomy, n^o^	29 (90,6%)
Lobectomy, n^o^	3 (9,4%)
Thyroid weight, grams	50,9 (±48,1)

A comparison between patients with and without incidental thyroid carcinoma is reported in Table 3.

**Table 3:** Comparison of demographic and clinical characteristics between patients with incidental thyroid carcinoma and those without carcinoma.

Variable	Carcinoma (*n* = 32)	No carcinoma (*n* = 191)	*p* value
Age, years (mean ± SD)	49.4 ± 16.7	56.2 ± 13.1	0.008
Female sex, *n* (%)	24 (75%)	157 (82.2%)	0.335
Male sex, *n* (%)	8 (25%)	34 (17.8%)	
Benign FNA (TIR2), *n* (%)	18 (56.3%)	133 (69.6%)	NS
No FNA performed, *n* (%)	18 (56.3%)	54 (28.3%)	NS
Thyroid weight, g (mean ± SD)	50.9 ± 48.1	71.3 ± 59.0	0.032
Total thyroidectomy, *n* (%)	29 (90.6%)	173 (90.6%)	NS
Lobectomy, *n* (%)	3 (9.4%)	18 (9.4%)	NS

Papillary thyroid carcinoma (PTC) was the most common type, identified in 25 cases (78.1%). Among these, 16 cases were papillary microcarcinomas (pT1a < 1 cm) and 9 cases were classic PTC. No aggressive variants (tall cell, hobnail, diffuse sclerosing) or follicular variants were identified. Follicular thyroid carcinoma was diagnosed in 5 cases (15.6%), while 1 case of anaplastic carcinoma and 1 case of oncocytic (Hürthle cell) carcinoma were identified, according to WHO 2022 classification.

The single case of anaplastic thyroid carcinoma was identified incidentally in a patient who underwent thyroidectomy for compressive multinodular goiter. Preoperative ultrasound showed a heterogeneous multinodular gland without specific features suggestive of aggressive malignancy, and FNAB had not been performed because no dominant suspicious nodules were identified. Histopathology revealed a focal anaplastic component arising in association with differentiated thyroid carcinoma.

**Figure 1:**
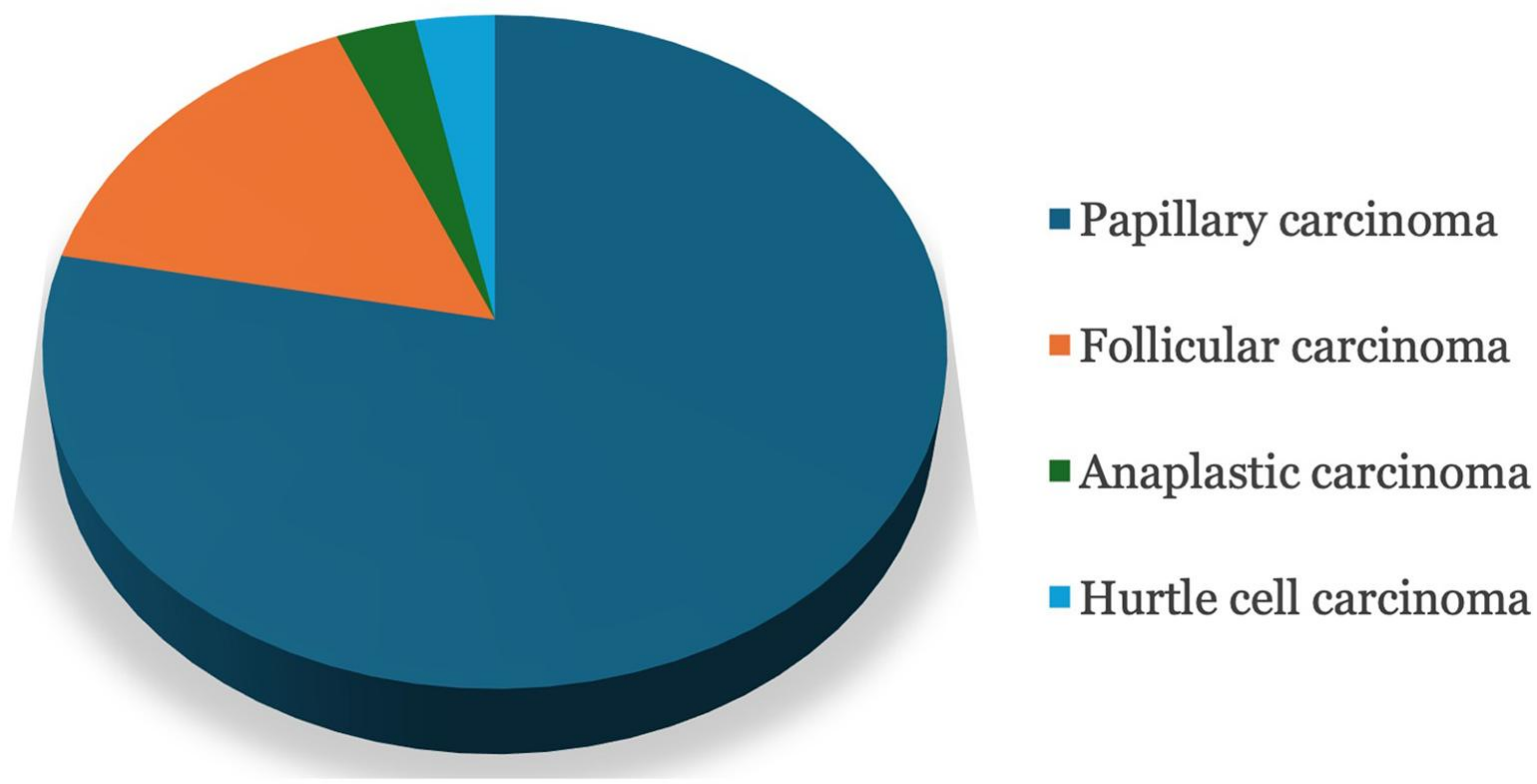
Subtypes of thyroid carcinoma.

No cases of medullary carcinoma were reported (Figure 1).

Regarding benign neoplasms, 20 follicular adenomas, 1 Hurthle cell adenoma, and 1 NIFTP (non-invasive follicular thyroid neoplasm with papillary-like nuclear features) were identified.

**Figure 2:**
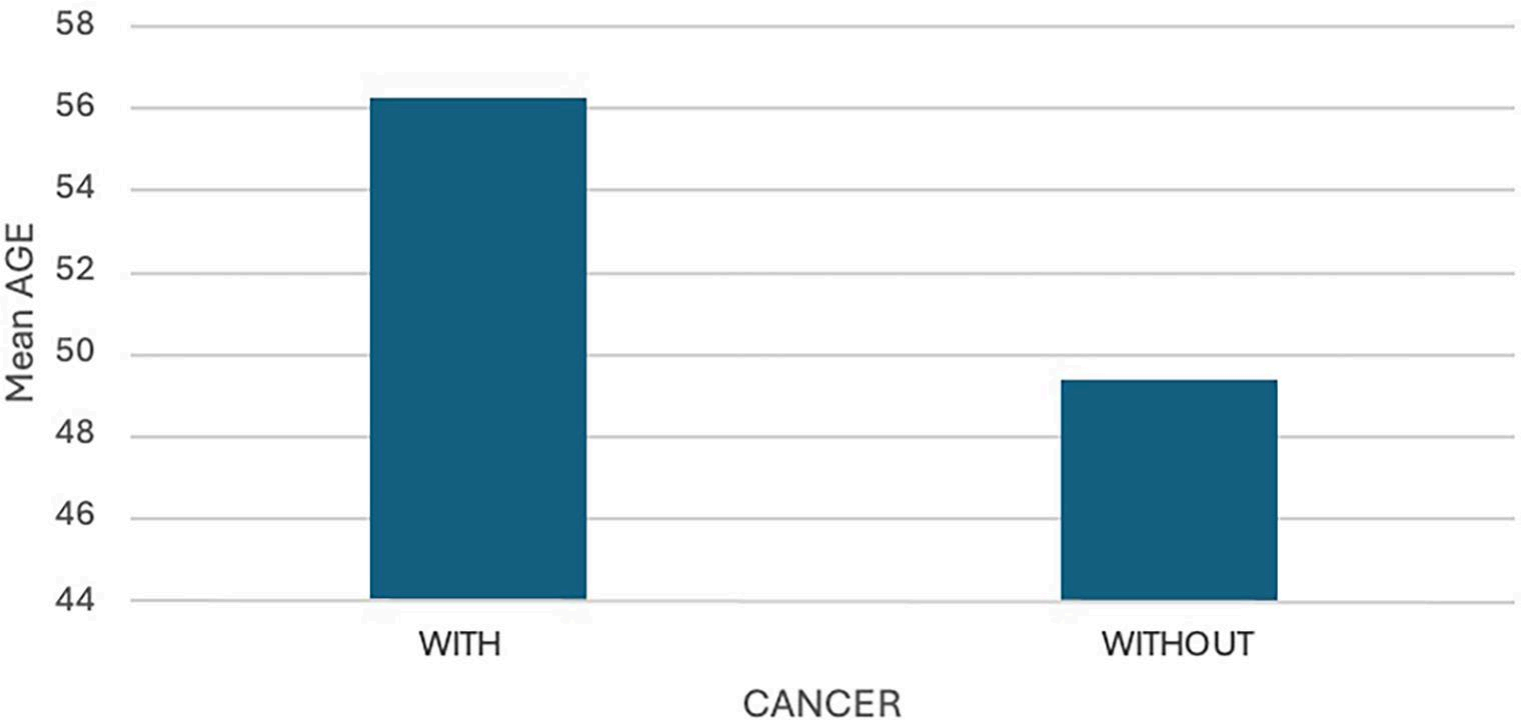
Age in the groups with an without carcinoma.

The mean age of patients with thyroid carcinoma was 49.4 years (SD 16.7), with an age range of 22–72 years. A significant difference was observed between this group and the mean age of patients without carcinoma (mean: 56.2 years, SD 13.1; Figure 2). Statistical analysis indicated a significant difference between the two groups in terms of age (t = −2.686; *p* = 0.008). Additionally, evaluating the correlation between age and malignancy revealed that for each additional year of age, the probability of carcinoma decreased by 3.7% (OR=0.963; *p* = 0.009).

No significant differences were found between the mean ages of male and female patients with carcinoma.

Following surgical removal, the weight of the thyroid glands was assessed as an indicative parameter of size, volume, and the density of the thyroid parenchyma, serving as a characteristic that integrates multiple structural factors of the organ affected by MNG.

**Figure 3:**
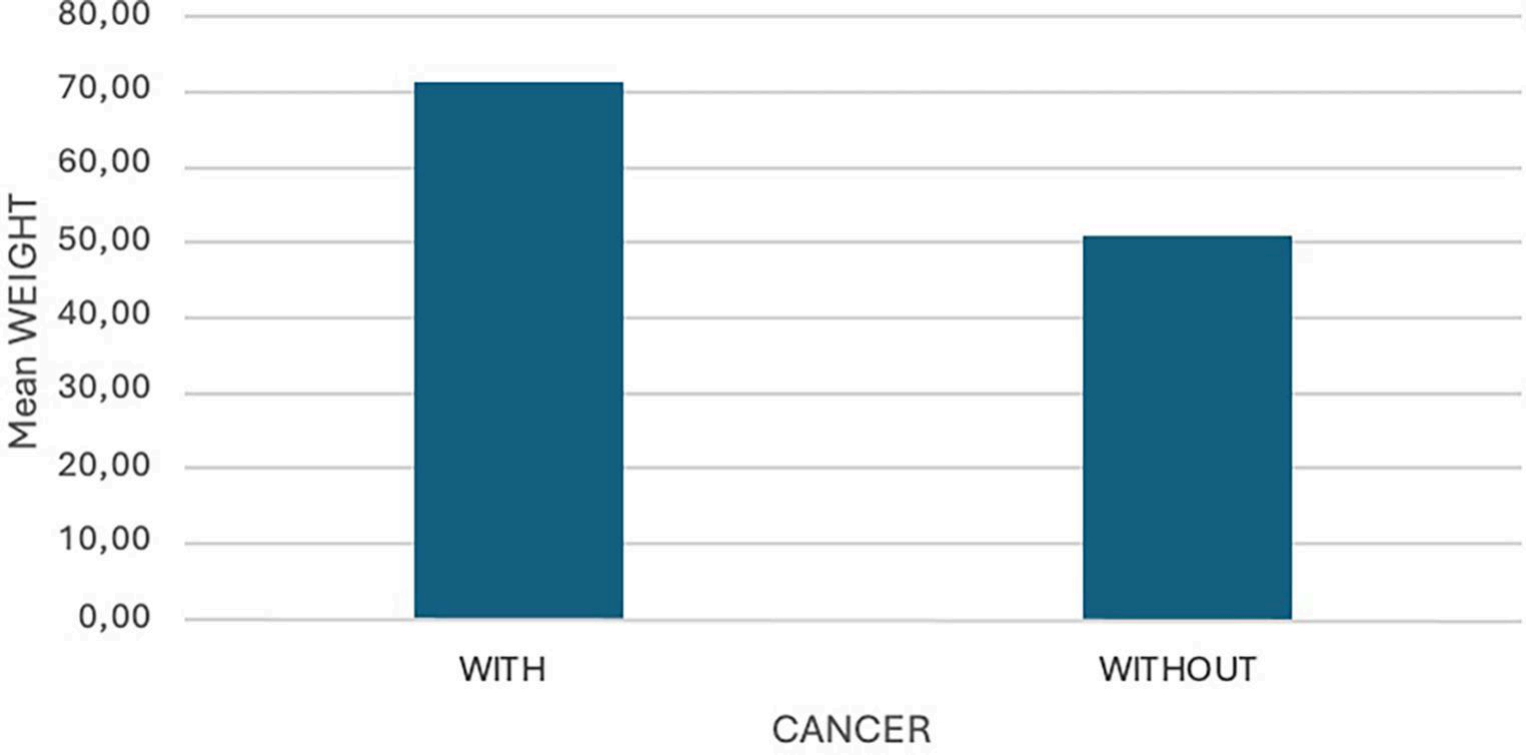
Weight in the groups with and without carcinoma.

The mean thyroid weight in patients with ITC was 50.9 grams (SD 48.1), compared to 71.3 grams (SD 59.0) in patients without ITC (Figure 3). This difference was statistically significant (t = −1.855; *p* = 0.032). No significant differences in carcinoma incidence were observed between patients with benign FNA results and those who did not undergo FNA.

## Discussion

In the present study, incidental thyroid carcinoma was identified in 14.4% of patients undergoing surgery for multinodular goiter. This finding confirms that malignant lesions may be detected with a non-negligible frequency in patients with MNG.

Our results are consistent with several studies conducted in Italian populations that have reported comparable incidence rates of incidental thyroid carcinoma in patients undergoing thyroidectomy for presumed benign disease (14, 15). Similar observations were described by Bove et al., Spaziani et al., and Chiefari et al (3, 16, 29). However, while these investigations reported a higher prevalence of carcinoma in female patients and the study by Apostolou et al. demonstrated a higher incidence among males (4), our analysis did not reveal statistically significant sex-related differences in cancer occurrence.

Regarding histological distribution, our findings confirm that papillary thyroid carcinoma represents the most common malignant subtype in patients with multinodular goiter, followed by follicular thyroid carcinoma. This pattern is consistent with numerous previous reports (2–6, 15).

It is worth noting that Hurthle cell carcinoma, previously defined according to the 2018 histopathological classification, has recently been reclassified as “oncocytic carcinoma” in the 2022 World Health Organization classification of thyroid tumors (17).

Approximately half of the papillary carcinomas identified in our cohort were microcarcinomas. Papillary thyroid microcarcinoma is generally associated with an excellent prognosis, with reported progression rates below 15% over a 10-year period (17, 18). The probability of progression appears to be higher in younger patients. Consequently, active surveillance with periodic ultrasonographic evaluation has been proposed as a safe management strategy in selected cases, particularly among older individuals, whereas younger patients may more frequently undergo surgical treatment. Nevertheless, several studies suggest that many young patients could also be managed conservatively without requiring surgery during their lifetime (19, 20).

The relationship between patient age and the risk of thyroid carcinoma in multinodular goiter remains debated. Some investigations have reported no significant association between age and malignancy (4, 21), whereas other studies—consistent with our findings—have suggested that younger patients may present a higher prevalence of thyroid cancer (22, 23).

In our cohort, 22 benign neoplasms were identified. These lesions typically require only conservative surgical treatment and generally do not necessitate radioactive iodine therapy or long-term oncological surveillance because of their low risk of progression.

Thyroid gland weight has also been proposed as a potential predictor of incidental thyroid carcinoma (4, 21, 24–26). Based on previous reports (4, 21), clinical observations, and diagnostic considerations related to nodular thyroid disease, we hypothesized that smaller thyroid glands—characterized by lower nodular density or more homogeneous parenchyma—may facilitate the detection of malignant lesions. In contrast, larger multinodular glands with complex structural architecture may hinder the identification of suspicious nodules during ultrasound examination or cytological sampling. Our results support this hypothesis, suggesting that thyroid weight could represent a useful parameter to consider during the clinical evaluation of patients with multinodular goiter (27, 28,29).

Several limitations of this study should be acknowledged. First, the relatively limited sample size and the single-center design may restrict the generalizability of the results. Second, a substantial proportion of patients did not undergo preoperative FNAB, reflecting the diagnostic challenges associated with multinodular goiter and the selective use of biopsy according to ultrasound findings. Third, complete preoperative ultrasound data—such as dominant nodule size and thyroid volume—were not consistently available because of the retrospective design of the study.

Future prospective multicenter studies incorporating standardized ultrasound parameters and larger patient populations will be necessary to better identify predictors of malignancy in patients with multinodular goiter.

## Conclusion

The present study confirms that incidental thyroid carcinoma is not uncommon in patients undergoing surgery for multinodular goiter. Papillary thyroid carcinoma was the predominant histological subtype, and no significant sex-related differences were observed. Although factors such as age and thyroid weight may contribute to risk stratification, the current evidence remains insufficient to modify existing screening or management strategies. Larger multicenter studies are warranted to identify reliable predictors of malignancy and improve the preoperative evaluation of patients with multinodular goiter.

## Data Availability

The raw data supporting the conclusions of this article will be made available by the authors, without undue reservation.
